# Analysis of the effects of early screening combined with blood lactate on the severity of patients with sepsis

**DOI:** 10.1016/j.heliyon.2024.e31907

**Published:** 2024-05-23

**Authors:** Qiyun Gan, Zhengning Li, Xin Li, Yinghua Huang, Haojian Deng

**Affiliations:** a Emergency Medicine Department, Liuzhou People's Hospital, Liuzhou, China; b General Practice, Liuzhou People's Hospital, Liuzhou, China; c Supply Room, Liuzhou People's Hospital, Liuzhou, China

**Keywords:** National early warning score, Blood lactic acid, Sepsis, Organ failure score, Early screening, Prognosis

## Abstract

This work aimed to investigate the adoption value of blood lactic acid (BLA) combined with the National Early Warning Score (NEWS) in the early screening of sepsis patients and assessing their severity. The data and materials utilized in this work were obtained from the electronic medical record system of 537 anonymized sepsis patients who received emergency rescue in the emergency rescue area of Liuzhou People's Hospital, Guangxi, from July 1, 2020, to December 26, 2020. Based on the 28-day outcomes of sepsis patients, the medical records were rolled into Group S (407 survival cases) and Group D (130 dead cases). Basic information such as the mode of hospital admission, initial management, use of emergency ventilator within 24 h of admission, NEWS score, arterial oxygen pressure/alveolar oxygen pressure ratio (PaO_2_/PAO_2_), alveolar-arterial oxygen difference (A-aDO_2_), serum creatinine (SCr), blood urea nitrogen (BUN), oxygenation index (OI), Glasgow Coma Scale (GCS), D-dimer, use of vasoactive drugs within 24 h of admission, C-reactive protein (CRP), procalcitonin (PCT), interleukin-6 (IL-6), N-terminal pro-B-type natriuretic peptide (NT-proBNP), quick Sequential Organ Failure Assessment (qSOFA) score, SOFA score, BLA level, NEWS with lactate (NEWS-L) score, SOFA score including lactate level (SOFA-L) score, Intensive Care Unit (ICU) length of stay, total hospital stay, ICU stay/total hospital stay, and septic shock condition were compared between groups. Logistic regression analysis was performed to assess the impact of various predictive factors on prognosis and to plot the receiver operating characteristic (ROC) curve. The results suggested marked differences between Group S and Group D in terms of mean age (*t* = −5.620; OR = −9.96, 95 % CI: −13.44∼-6.47; *P* < 0.001). Group S showed drastic differences in terms of mode of hospital admission (χ^2^ = 9.618, *P* < 0.01), method of initial management (χ^2^ = 51.766, *P* < 0.001), use of emergency ventilator within 24 h of admission (χ^2^ = 98.564, *P* < 0.001), incidence of septic shock (χ^2^ = 77.545, *P* < 0.001), use of vasoactive drugs within 24 h of admission (χ^2^ = 102.453, *P* < 0.001), heart rate (*t* = −4.063, *P* < 0.001), respiratory rate (*t* = −4.758, *P* < 0.001), oxygenation status (χ^2^ = 20.547, *P* < 0.001), NEWS score (*t* = −6.120, *P* < 0.001), PaO_2_/PAO_2_ ratio (*t* = 2.625, *P* < 0.01), A-aDO_2_ value (*Z* = −3.581, *P* < 0.001), OI value (*Z* = −3.106, *P* < 0.01), PLT value (*Z* = −2.305, *P* < 0.05), SCr value (*Z* = −3.510, *P* < 0.001), BUN value (*Z* = −3.170, *P* < 0.01), D-dimer (*Z* = −4.621, *P* < 0.001), CRP level (*Z* = −4.057, *P* < 0.001), PCT value (*Z* = −2.783, *P* < 0.01), IL-6 level (*Z* = −2.904, *P* < 0.001), length of hospital stay (*Z* = −4.138, *P* < 0.001), total hospital stay (*Z* = −8.488, *P* < 0.001), CCU/total hospital stay (*Z* = −9.118, *P* < 0.001), NEWS score (*t* = −6.120, *P* < 0.001), SOFA score (*t* = −6.961, *P* < 0.001), SOFA-L score (*Z* = −4.609, *P* < 0.001), NEWS-L score (*Z* = −5.845, *P* < 0.001), BLA level (*Z* = −6.557, *P* < 0.001), and GCS score (*Z* = 6.909, *P* < 0.001) when compared to Group D. The use of ventilators, septic shock, PCT, NEWS score, GCS score, SOFA score, SOFA-L score, NEWS-L score, and BLA level were identified as independent risk factors for predicting the prognosis of sepsis patients (*P* < 0.001). The areas under ROC curve (AUC) of blood lactic acid, PCT, NEWS, NEWS-L, GCS, SOFA, and SOFA-L were 0.695, 0.665, 0.692, 0.698, 0.477, 0.700, and 0.653, respectively. These findings indicate that the combination of BLA with NEWS (NEWS-L) score and SOFA score has certain advantages in assessing the prognosis of sepsis.

## 1 Introduction

Sepsis is a systemic inflammatory response syndrome (SIRS) caused by microbial infection. Many inflammatory mediators are produced in patients' bodies, which activate coagulation and fibrinolysis systems, and tissue perfusion is insufficient. If not treated in time, it may lead to multiple organ dysfunction syndrome (MODS) with high mortality. It is estimated that the annual incidence of sepsis in developed countries is increasing at a rate of 8 %–13 %, with approximately 4 million people dying from sepsis worldwide each year [1]. The overall prevalence rate of sepsis in Asian intensive care units (ICUs) is as high as 22.4 %, of which the incidence rate and mortality rate in low and middle-income countries and regions are as high as 24.5 % and 32.6 % [2]; The incidence rate of sepsis in China is 421.85/100,000, and its incidence rate is increasing year by year, of which 57.5 % are elderly people over 65 years old [3]. Sepsis has a rapid and insidious onset, triggering a systemic inflammatory response and potentially leading to multiorgan dysfunction. Simultaneously, sepsis can cause severe metabolic disturbances, such as acidosis and electrolyte imbalances, posing further threats to the patient's overall physiological and psychological well-being [4]. Current research indicates that septic patients are prone to serious complications, including septic shock and multiple organ dysfunction, which are associated with high mortality rates, particularly among bedridden or elderly patients [5]. Sepsis leads to prolonged treatment duration and increased mortality rates in critical care units such as the Intensive Care Unit (ICU), imposing remarkable impacts on patients, families, and society [6]. Hence, early and proactive diagnosis is of great significance for clinical guidance, intervention selection, and prognosis in septic patients.

Numerous studies have demonstrated that determining the severity of sepsis based on patient-related indicators in the early stages is an effective approach to improving survival rates and reducing complications in septic patients. blood lactic acid (BLA) is utilized to assess tissue perfusion and cellular dysfunction, reflecting tissue hypoxia and hemodynamic changes. Several clinical studies have confirmed a close association between high BLA levels and mortality rate and prognosis in septic patients [7,8], highlighting its value in assessing the severity of sepsis. Elevated levels of procalcitonin (PCT) and C-reactive protein (CRP) are commonly observed in sepsis. Dynamic monitoring of arterial blood lactate, PCT, and CRP levels plays a crucial role in improving the prognosis of septic patients, enhancing their quality of life, and increasing survival rates. Researchers have focused on septic pediatric patients to evaluate the relationship between blood lactate levels, PCT, CRP, and the severity and prognosis of neonatal sepsis. The results revealed a significant negative correlation between blood lactate levels and neonatal critical illness score (NCIS), a significant positive correlation among blood lactate, PCT, and CRP, and a significant negative correlation between PCT, CRP, and NCIS scores. The levels of CRP and PCT in septic children were higher than those in the control group. There exists a significant correlation among blood lactate, CRP, PCT, and NCIS. Lower NCIS scores corresponded to more significant increases in blood lactate, PCT, and CRP. This indicates that combined monitoring of blood lactate, PCT, and CRP levels can predict the severity and prognosis of neonatal sepsis patients. While high BLA levels are a sensitive indicator, it should be noted that various other conditions, such as tumors, liver dysfunction, heart failure, anemia, and toxic shock, can also cause elevated lactate levels [9]. Furthermore, due to individual variations and differences in laboratory testing methods and standards, there is a certain degree of measurement error associated with BLA levels. Hence, when using BLA as an indicator, it is necessary to consider the patient's clinical presentation and other parameters in a comprehensive manner. Currently, other tools utilized for sepsis assessment include the National Early Warning Score (NEWS), quick Sequential Organ Failure Assessment (qSOFA), Sequential Organ Failure Assessment (SOFA), National Early Warning Score (NEWS), SOFA with lactate (SOFA-L), and Glasgow Coma Scale (GCS) [10]. The NEWS is designed to improve the accurate assessment of patient acuity and facilitate rapid intervention, thus reducing the occurrence of adverse outcomes. In the diagnosis of sepsis, the NEWS score can rapidly reflect the severity of the patient's condition and changes in their clinical status, aiding physicians in promptly adjusting treatment plans. Additionally, the NEWS score can predict the risk of adverse outcomes in septic patients, including mortality, the need for intensive care and/or mechanical ventilation, thereby increasing awareness of patient risk and helping physicians implement more precise interventions to improve treatment efficacy and prognosis. Nevertheless, the NEWS score has certain limitations in sepsis diagnosis. It does not encompass all relevant factors, and the weighting of individual parameters may not be universally applicable. Furthermore, its effectiveness in specific populations is lacking [11]. Hence, there are still some inherent limitations in the diagnostic adoption of the NEWS in sepsis.

Taking into account the aforementioned relevant research literature, NEWS and BLA have their respective advantages and limitations in screening and prognostic assessment of septic patients. Nevertheless, there is currently a lack of detailed research on the use of these two indicators for early screening. Hence, the relevant information from the anonymized electronic medical records of 536 septic patients treated in the emergency rescue area of Liuzhou People's Hospital in Liuzhou City, Guangxi, from July 1, 2020, to December 26, 2020, was utilized as the data source. This hospital is a regional medical center with 1500 beds, and its emergency department receives approximately 2,000,000 patients annually, with the emergency rescue area handling approximately 25,000 patients each year. The aim was to investigate the combined use of the NEWS and BLA for early screening of the severity of septic patients, in order to provide scientific evidence for the early identification and intervention of sepsis, improve the survival rate and prognosis of septic patients, and provide data support for the revision of clinical diagnostic guidelines. Furthermore, this work aims to promote the use of novel screening tools in clinical practice and provide references for the precision treatment of sepsis.

## 2 Materials and methods

### 2.1 Research data

The data and materials for this work were obtained from the electronic medical records system of the Emergency Rescue Area at Liuzhou People's Hospital in Liuzhou City, Guangxi, which were retrospectively collected from July 1, 2020, to December 26, 2020. A total of 537 cases of anonymized septic patients was collected. Among them, there were 332 males (61.82 %) and 205 females (38.18 %), with an age range of 15–99 years and a mean age of 62.23 ± 18.01 years. The access time of the electronic medical record system for this study was June 30, 2021, and the procedure of this study had been approved by the Medical Ethics Committee of Liuzhou People's Hospital (approval number: No.201932H). Inclusion criteria were as follows: (1) patients diagnosed with sepsis according to the sepsis 3.0 diagnostic criteria [12]: life-threatening organ dysfunction resulting from host response dysregulation in the context of infection can be indicated by an increase in the SOFA score of >2; septic shock is defined as systemic infection accompanied by sustained hypotension, requiring vasopressor therapy to maintain a mean arterial pressure ≥65 mmHg despite adequate fluid resuscitation, along with serum lactate levels >2 mmol/L (>18 mg/dL); (2) patients having complete recorded data in the electronic medical records. Exclusion criteria were as follows: (1) patients with concomitant malignant tumors or hematological disorders; (2) pregnant or lactating women; (3) patients undergoing treatment with metformin; (4) patients with autoimmune diseases; (5) patients with central nervous system diseases; (6) patients with hepatic or renal insufficiency; (7) patients who died within 24 h of admission.

### 2.2 Grouping and NEWS evaluation methodologies

Based on the 28-day outcomes of septic patients, the medical records were rolled into group S (407 survival cases) and group D (130 dead cases).

The NEWS system assessed the following vital signs parameters: respiratory rate, blood pressure, oxygen saturation (SpO_2_), whether oxygen therapy was administered during SpO_2_ measurement, heart rate, body temperature, and level of consciousness. Each parameter was assigned a specific score, resulting in a total score of 20. Different score ranges were interpreted as increasing levels of risk. Scores of 1–2 were considered normal or low risk, scores of 3–4 were classified as moderate risk, and scores of 5 or above were considered high risk. The specific evaluation criteria for the NEWS system are presented in Table 1.

**Table 1:** NEWS evaluation standard.

Score	Body temperature (°C)	Respiratory frequency (times/min)	Heart rate (beats/min)	Systolic blood pressure (SBP, mmHg)	Consciousness state	SpO_2_ (%)	Oxygen inhalation
3 points	≤35.0	≤8 or ≥25	≤40 or ≥131	≤90 or ≥220	V/P/U	≤91	
2 points	≥39.1	21∼24	111∼130	91∼100		92∼93	Yes
1 point	35.1–36.0 or 38.1–39.0	9∼11	41∼50 or 91∼110	101∼110		94∼95	
0 point	36.1–38.0	12∼20	51∼90	111∼219	A	≥96	No

Remarks: A is conscious, V is responsive to sound, P is responsive to pain, and U is unresponsive.

### 2.3 Observation indexes

(1) Demographic information included in the analysis comprised age, gender, mode of admission, hospital disposition, medical history, underlying comorbidities, and site of infection within 48 h of admission.
(2) Clinical indicators included in the analysis were as follows: NEWS score, SBP, diastolic blood pressure (DBP), arterial pH value, partial pressure of oxygen in arterial blood (PO_2_), partial pressure of carbon dioxide in arterial blood (PCO_2_), base excess, ratio of arterial oxygen partial pressure to fractional inspired oxygen (PaO_2_/PAO_2_), arteriovenous oxygen difference (A-aDO_2_), hemoglobin (Hb), hematocrit (Hct), platelets (PLT), mean arterial pressure (MAP), creatinine (SCr), blood urea nitrogen (BUN), oxygenation index (OI), total bilirubin (TBIL), GCS, D2 dimer, and use of vasoactive drugs within 24 h of admission.
(3) Infection biomarkers included CRP, PCT, interleukin-6 (IL-6), and N-terminal pro-B-type natriuretic peptide (NT-proBNP).
(4) Variables assessed within 24 h of admission included the use of emergency mechanical ventilation, 28-day outcomes, qSOFA score, SOFA score, BLA levels, National early warning score combined with blood lactic acid (NEWS-L) score, and SOFA score combined with blood lactic acid (SOFA-L) score.
(5) Hospitalization details encompassed ICU length of stay, total length of hospitalization, and ICU stay as a proportion of the total hospitalization duration.
(6) Statistical analysis of septic shock cases was conducted.

### 2.4 Statistical methodologies

Using SPSS 22.0, for normally distributed continuous data with equal variances, the mean ± standard deviation ($\bar{x}$ ±s) was reported, and independent samples *t*-test was utilized for between-group comparisons. For non-normally distributed continuous data, the median (Q_1_, Q_3_) was reported, and the Mann-Whitney *U* test was utilized for between-group comparisons. Categorical data were presented as percentages (%), and the chi-square test was utilized for analysis. Logistic regression analysis was conducted to assess the impact of various predictor variables on the outcome. Receiver operating characteristic (ROC) curves were plotted, and the area under the ROC curve with a 95 % confidence interval (CI) was calculated to evaluate the predictive ability for 28-day mortality. An area under the ROC curve of 1.0 indicates perfect discrimination, while an area under 0.5 indicates no discrimination. The optimal cutoff point was determined using the Youden index (YI), and sensitivity (Sen) and specificity (Spe) of each variable were calculated. Statistically, *P* < 0.05 was deemed significant for all analyses.

## 3 Results

### 3.1 General data analysis

**Fig. 1:**
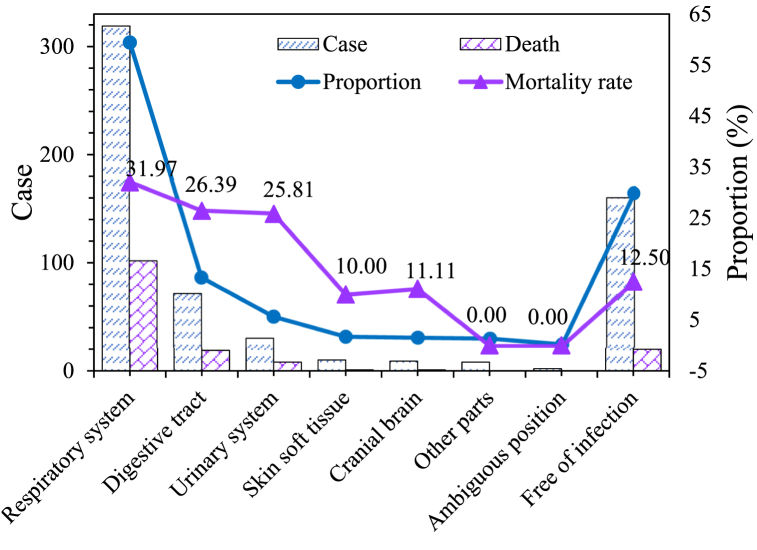
Statistics of infection site and mortality rate.

A statistical analysis was conducted on the infected sites and mortality rates among the included cases in this work (Fig. 1). The respiratory system was the most common site of infection, with a total of 319 cases (59.40 %). There were 72 cases (13.41 %) of abdominal and digestive tract infections, 31 cases (5.77 %) of urinary system infections, 10 cases (1.86 %) of skin and soft tissue infections, and 9 cases (1.68 %) of central nervous system infections. Other sites (including bone marrow, pericardium, myocardium, endocardium, and bloodstream) accounted for 8 cases (1.49 %). In 2 cases (0.37 %), the infection site was not clearly identified. Additionally, 160 cases (29.80 %) did not have any documented infection. The statistical analysis of mortality rates based on various infection sites revealed that among the 130 deceased cases, 102 (31.97 %) had respiratory system infections and died. In the cases with abdominal and digestive tract infections, 19 (26.39 %) resulted in death, while 8 (25.81 %) of those with urinary system infections and 1 (10.00 %) of those with skin and soft tissue infections experienced mortality. Similarly, among the cases with central nervous system infections, 1 (11.11 %) resulted in death. No deaths were reported among cases with infections in other sites or cases with unidentified infection sites. Among the cases without documented infections, 20 (12.50 %) deaths occurred.

### 3.2 Comparison of basic clinical data

Table 2 presents the basic characteristics of Group S and Group D cases. The sex distribution differed substantially between groups (χ^2^ = 5.799, *P* < 0.05). There was an extremely marked difference in the average age between groups (*t* = −5.620; OR = −9.96; 95 % CI: −13.44∼-6.47; *P* < 0.001). In terms of the mode of arrival, group S had a higher proportion of self-arrival, while group D had a higher proportion of arrival by ambulance (χ^2^ = 9.618, *P* < 0.01). Regarding the admission disposition, group S primarily received general treatment, while group D had a higher proportion of ICU admission (χ^2^ = 51.766, *P* < 0.001). No great differences (*P* > 0.05) were found between groups D and S in terms of medical history of hypertension, chronic obstructive pulmonary disease, diabetes, and heart disease. The statistical results of emergency ventilator use within 24 h of admission showed that group D had a higher proportion of invasive ventilator use, while the majority of group S (69.29 %) did not require ventilator support. In group D, the proportion of invasive ventilator use was significantly higher at 79.23 % (103/130) compared to group S, which had a proportion of 30.22 % (123/407). The emergency ventilator use within 24 h of admission differed considerably between groups (χ^2^ = 98.564, *P* < 0.001). Regarding the occurrence of septic shock during hospitalization, the statistical results showed that the incidence of septic shock in group S during hospitalization was 5.65 %, drastically inferior to the 35.38 % in group D (χ^2^ = 77.545, *P* < 0.001). The statistical analysis of the use of vasoactive drugs within 24 h of admission revealed that group S had 44 cases (10.81 %) of vasoactive drug use, which was drastically inferior to the 68 cases (52.31 %) in group D. An extremely notable difference existed in the use of vasoactive drugs within 24 h of admission between groups (χ^2^ = 102.453, *P* < 0.001).

**Table 2:** Comparison of basic data of included objects.

Factor	Group S (n = 407)	Group D (n = 130)	*χ*^*2*^*/t*	*P*
Sex [case (%)]			5.799	0.016*
Male	275 (67.57 %)	57 (43.80 %)		
Female	132 (32.43 %)	73 (56.20 %)		
Age (years old)	59.76 ± 18.47	69.72 ± 14.43	−5.620	<0.001***
Mode of admission			9.618	0.002**
By oneself	235 (57.74 %)	55 (42.30 %)		
Ambulance	172 (42.26 %)	75 (57.70 %)		
Admission disposal methods			51.766	<0.001***
ICU	153 (37.59 %)	96 (73.80 %)		
Common	254 (62.41 %)	34 (26.20 %)		
Past medical history				
Hypertension	105 (25.90 %)	45 (34.60 %)	3.744	0.053
Chronic obstructive pulmonary disease	13 (3.20 %)	2 (1.50 %)	1.002	0.317
Diabetes	56 (13.80 %)	23 (17.70 %)	1.191	0.275
Heart disease	50 (12.30 %)	16 (12.30 %)	0.000	0.998
Emergency ventilator was used			98.564	<0.001***
Invasive	123 (30.22 %)	103 (79.23 %)		
Noninvasive	2 (0.49 %)	1 (0.77 %)		
No	282 (69.29 %)	26 (20.00 %)		
Septic shock			77.545	<0.001***
Yes	23 (5.65 %)	46 (35.38 %)		
No	384 (94.35 %)	84 (64.62 %)		
Use of vasoactive agents			102.453	<0.001***
Yes	44 (10.81 %)	68 (52.31 %)		
No	363 (89.19 %)	62 (47.69 %)		

*P < 0.05, **P < 0.01, ***P < 0.001 vs. group D.

### 3.3 Comparison of two groups of NEWS scoring system indicators

Table 3 presents the statistical results of NEWS scoring system-related indicators and NEWS scores in group S and group D cases. Slight differences (*P* > 0.05) were indicated between groups in terms of average body temperature, blood WBC count, and SBP. The group S had greatly inferior average heart rate (*t* = −4.063, *P* < 0.001) and average respiratory rate (*t* = −4.758, *P* < 0.001) to group D. The percentage of cases receiving oxygen therapy in group S (146, 36.00 %) was drastically inferior to in group D (260, 64.00 %), showing an extremely marked difference in oxygen therapy status between groups (χ^2^ = 20.547, *P* < 0.001). Regarding the level of consciousness, inconsiderable differences (*P* > 0.05) existed in the proportion of P and V cases between groups. The proportion of U cases in group S was inferior to group D (χ^2^ = 35.856, *P* < 0.001), while the proportion of A cases in group S was superior to group D (χ^2^ = 23.294, *P* < 0.001). The average NEWS score in group S was 8.61 ± 2.93, drastically inferior to the average NEWS score of 11.10 ± 3.428 in group D, implying dramatic difference in the average NEWS scores between groups (*t* = −6.120, *P* < 0.001).

**Table 3:** Comparison of two groups of NEWS scoring system indicators.

Factor	Group S (n = 407)	Group D (n = 130)	*χ*^*2*^*/t*	*P*
Body temperature (°C)	36.87 ± 0.92	36.97 ± 0.89	−1.096	0.273
Heart rate (beats/min)	93.29 ± 22.52	103.00 ± 27.14	−4.063	<0.001***
Respiratory rate (breaths/min)	23.95 ± 3.16	25.64 ± 4.45	−4.758	<0.001***
WBC ( × 10^9^/L)	12.30 ± 6.21	12.77 ± 6.08	−0.760	0.448
SpO_2_ (%)	95.10 ± 6.98	91.73 ± 9.75	3.903	<0.001***
Oxygen inhalation			20.547	<0.001***
Yes	146 (35.87 %)	76 (58.46 %)		
No	261 (64.13 %)	54 (41.54 %)		
SBP (mmHg)	115.67 ± 37.27	123.22 ± 49.34	−1.845	0.066
State of consciousness				
U	60 (14.80 %)	51 (39.20 %)	35.856	<0.001***
P	45 (11.10 %)	16 (12.30 %)	0.146	0.702
V	104 (25.60 %)	34 (26.20 %)	0.015	0.903
A	184 (45.30 %)	28 (21.50 %)	23.294	<0.001***
NEWS score	8.61 ± 2.93	11.10 ± 3.42	−6.120	<0.001***

***P < 0.001 vs. group D.

### 3.4 Comparison of clinical examination indexes

Table 4 presents the statistical results of clinical examination indicators in group S and group D cases. Neglectable differences (*P* > 0.05) was suggested between groups in terms of DBP, PO_2_, PCO_2_, and MAP. The group S exhibited superior values of PaO_2_/PAO_2_ to group D (*t* = 2.625, *P* < 0.01). The group S showed remarkably inferior levels of A-aDO_2_ to group D (*Z* = −3.581, *P* < 0.001). The group S had higher levels of OI versus group D (*Z* = −3.106, *P* < 0.01).

**Table 4:** Comparison of clinical examination indexes.

Factor	Group S (n = 407)	Group D (n = 130)	*t/Z*	*P*
DBP (mmHg)	69.29 ± 20.22	71.00 ± 25.50	−0.786	0.432
PO_2_ (mmHg)	100.09 ± 41.40	98.26 ± 47.21	0.368	0.713
PCO_2_ (mmHg)	34.45 ± 12.34	35.05 ± 14.22	0.265	0.791
MAP (mmHg)	84.75 ± 25.35	88.62 ± 132.30	−1.414	0.158
PaO_2_/PAO_2_	0.75 ± 0.30	0.64 ± 0.39	2.625	0.009**
A-aDO_2_	29.00 (14.75,70.00)	49.00 (23.00,127.00)	−3.581	<0.001***
OI (mmHg)	381.0 (267.00,471.25)	310.0 (185.00,456.00)	−3.106	0.002**

**P < 0.01, ***P < 0.001 vs. group D.

### 3.5 Comparison of blood test indexes

Table 5 presents the statistical results of blood test indicators in group S and group D cases. Inconsiderable differences (*P* > 0.05) existed between groups in terms of Hb, Hct, pH value, base excess, and TBIL. The group S exhibited higher values of PLT than group D (*Z* = −2.305, *P* < 0.05). The group S showed drastically lower levels of SCr versus group D, with an extremely marked difference (*Z* = −3.510, *P* < 0.001). The group S had inferior levels of BUN to group D (*Z* = −3.170, *P* < 0.01), and the group S displayed lower levels of D2 dimer relative to group D (*Z* = −4.621, *P* < 0.001).

**Table 5:** Comparison of blood test indexes.

Factor	Group S (n = 407)	Group D (n = 130)	*t/Z*	*P*
Hb (g/L)	112.09 ± 29.72	107.51 ± 31.56	1.507	0.132
Hct (%)	33.69 ± 8.45	32.53 ± 9.42	1.328	0.185
PLT ( × 10^9^/L)	214.50 (160.75,272.0)	191.0 (134.25,248.5)	−2.305	0.021*
pH	7.43 (7.39,7.47)	7.36 (7.36,7.47)	−1.791	0.073
Base excess (mmol/L)	−0.80 (−4.30,1.50)	−1.10 (−8.65,1.80)	−1.095	0.274
SCr (μmol/L)	79.50 (64.15,111.70)	93.20 (67.50,173.0)	−3.510	<0.001***
BUN (mg/dL)	6.25 (4.36,9.90)	7.67 (4.96,15.92)	−3.170	0.002**
TBIL (μmol/L)	12.30 (8.30,19.25)	13.20 (9.00,23.00)	−1.278	0.201
D2 dimer (mg/L)	1.58 (0.59,4.42)	2.77 (1.60,5.23)	−4.621	<0.001***

*P < 0.05, **P < 0.01, ***P < 0.001 vs. group D.

### 3.6 Comparison of infection markers

The results of the normality test indicated that the infection biomarker-related indicators in both groups did not follow a normal distribution. Hence, non-parametric tests were utilized for their analysis. The detection results of the infection biomarker-related indicators in both groups are presented in Table 6. The group S (sepsis) exhibited considerably inferior levels of CRP to group D (non-sepsis) (*Z* = −4.057, *P* < 0.001). The group S also showed lower levels of PCT than group D (*Z* = −2.783, *P* < 0.01). Furthermore, the group S demonstrated greatly lower levels of IL-6 relative to group D (*Z* = −2.904, *P* < 0.001). The group S displayed lower levels of NT-proBNP versus group D, but the difference was inconsiderable in NT-proBNP levels between groups (*Z* = −1.715, *P* = 0.086).

**Table 6:** Comparison of infection markers.

Factor	Group S (n = 407)	Group D (n = 130)	*Z*	*P*
CRP (mg/L)	56.73 (9.07,122.07)	140.07 (46.18,163.64)	−4.057	<0.001***
PCT (pg/mL)	0.43 (0.15,1.47)	2.06 (1.46,4.70)	−2.783	0.005**
IL-6 (pg/mL)	50.39 (29.39,93.76)	203.30 (61.67,341.50)	−5.904	<0.001***
NT-proBNP (pg/mL)	2550 (593.03,6409.75)	3920 (2359.00,6613.00)	−1.715	0.086

**P < 0.01, ***P < 0.001 vs. group D.

### 3.7 Comparison of hospitalization time

The results of the normality test indicated that the hospitalization durations in both groups did not follow a normal distribution. Hence, non-parametric tests were utilized for their analysis. The statistical results of the hospitalization durations in both groups are presented in Table 7. The group S exhibited notably superior values for ICU stay and total hospital stay to group D (*P* < 0.001). Additionally, the group S showed greatly lower ICU/total hospital stay versus group D (*Z* = −9.118, *P* < 0.001).

**Table 7:** Comparison of hospitalization time.

Factor	Group S (n = 407)	Group D (n = 130)	*Z*	*P*
ICU stay (d)	9.00 (6.00,14.00)	6.00 (2.00,12.00)	−4.137	<0.001***
Total length of stay (d)	18.00 (11.00,29.00)	6.00 (3.00,12.25)	−8.488	<0.001***
ICU/Total length of stay (%)	52.90 (35.45,100.00)	100.00 (80.56,100.00)	−9.118	<0.001***

***P < 0.001 vs. group D.

### 3.8 Comparison of early screening indexes

The results of the normality test indicated that the NEWS score, GCS score, and NEWS-L score in both groups of early screening indicators followed a normal distribution. Hence, independent samples *t*-test was utilized for the analysis of these variables. Additionally, the SOFA score, SOFA-L score, and BLA level did not follow a normal distribution. Hence, non-parametric tests were employed for their analysis. The statistical results of the early screening indicators in both groups are presented in Table 8. The group S demonstrated inferior values for NEWS score, SOFA score, SOFA-L score, NEWS-L score, and BLA level to group D (non-sepsis) (*P* < 0.001). Additionally, the group D exhibited superior GCS score to group S (*Z* = 6.909, *P* < 0.001).

**Table 8:** Comparison of sepsis screening indexes.

Factor	Group S (n = 407)	Group D (n = 130)	*t/Z*	*P*
NEWS score	8.61 ± 2.93	11.10 ± 3.42	−6.120	<0.001***
GCS score	12.16 ± 3.80	9.38 ± 4.48	6.909	<0.001***
SOFA score	4.00 (2.00,13.00)	8.00 (5.00,10.50)	−6.961	<0.001***
SOFA-L score	2.10 (1.20,3.30)	2.90 (1.60,4.90)	−4.609	<0.001***
NEWS-L score	11.43 ± 4.51	15.09 ± 5.24	−5.845	<0.001***
BLA (mmol/L)	6.25 (4.40,10.15)	11.40 (7.60,15.10)	−6.557	<0.001***

***P < 0.001 vs. group D.

### 3.9 Analysis of independent risk factors of sepsis death

Univariate analysis revealed several notable factors influencing mortality in sepsis, including gender, age, mode of admission, initial management at admission, emergency ventilator use, septic shock, vasopressor use, heart rate, respiratory rate, SpO_2_, oxygen supplementation, PaO_2_/PAO_2_ ratio, A-aDO_2_, OI, PLT count, SCr level, BUN level, D2 dimer, CRP level, PCT level, IL-6 level, ICU admission, total hospital stay, ICU/total hospital stay, NEWS score, GCS score, SOFA score, SOFA-L score, NEWS-L score, and BLA level. Logistic multivariable regression analysis was performed using these factors as independent variables to determine their association with sepsis mortality (Table 9). Ventilator use, septic shock, PCT level, NEWS score, GCS score, SOFA score, SOFA-L score, NEWS-L score, and BLA level were all closely associated with sepsis mortality (*P* < 0.001). Hence, ventilator use, septic shock, PCT level, NEWS score, GCS score, SOFA score, SOFA-L score, NEWS-L score, and BLA level emerged as independent risk factors for predicting the prognosis of septic patients.

**Table 9:** Analysis of risk factors related to sepsis mortality by binary Logistic regression.

Factor	Β coefficient	Standard error	Wald χ^2^	OR	95%CI	*P*
Age	0.022	0.013	2.748	1.022	0.996–1.048	0.097
Sex	21.070	97.421	0.104	14.141	0.486–25.486	0.808
Mode of admission	19.462	50.052	0.087	23.18	0.293–41.25	0.799
Admission disposal methods	−1.218	10.030	0.724	0.296	0.024–20.34	0.695
Heart rate	0.009	0.008	1.384	1.010	0.994–1.026	0.239
Respiratory rate	0.067	0.077	0.750	1.069	0.919–1.243	0.386
Use of ventilator	−3.595	1.097	10.742	0.027	0.003–0.236	<0.001***
SPO_2_	0.002	0.035	0.002	1.002	0.935–1.073	0.960
PaO_2_/PAO_2_	0.090	2.096	0.002	1.094	0.018–66.497	0.966
A-aDO_2_	0.002	0.005	0.147	1.002	0.993–1.011	0.701
OI	0.001	0.003	0.045	1.001	0.995–1.007	0.833
Septic shock	−1.540	0.473	10.596	0.214	0.085–0.542	<0.001***
Use of vasoactive agents	−20.037	38.507	0.017	0.751	0.053–8.576	0.696
SCr	0.003	0.004	0.503	1.003	0.994–1.012	0.487
PLT	−0.005	0.004	1.216	0.995	0.995–0.987	0.270
BUN	0.053	0.065	0.653	1.054	0.928–1.197	0.419
D2 dimer	0.012	0.105	0.013	1.012	0.824–1.243	0.910
CRP	0.008	0.008	0.926	1.008	0.992–1.024	0.336
PCT	0.068	0.079	9.742	1.371	1.016–1.851	<0.001***
IL-6	0.001	0.002	0.438	1.001	0.998–1.004	0.508
ICU stay	−0.077	0.193	0.157	0.926	0.634–1.353	0.692
Total length of stay	−0.023	0.171	0.018	0.977	0.699–1.365	0.892
CCU/Total length of stay	0.038	0.029	1.642	1.038	0.980–1.100	1.038
NEWS score	0.151	0.468	20.376	1.143	1.004–1.236	<0.001***
NEWS-L score	0.246	0.215	59.26	1.896	1.563–2.320	<0.001***
GCS score	0.033	0.036	102.53	1.968	1.303–3.037	<0.001***
SOFA score	0.067	0.246	98.830	1.936	1.578–3.016	<0.001***
SOFA-L score	0.508	0.402	76.543	1.602	1.274–2.363	<0.001***
BLA	0.293	0.247	21.252	1.276	1.038–3.094	<0.001***

### 3.10 Evaluation of 28-day prognosis by BLA combined index

**Fig. 2:**
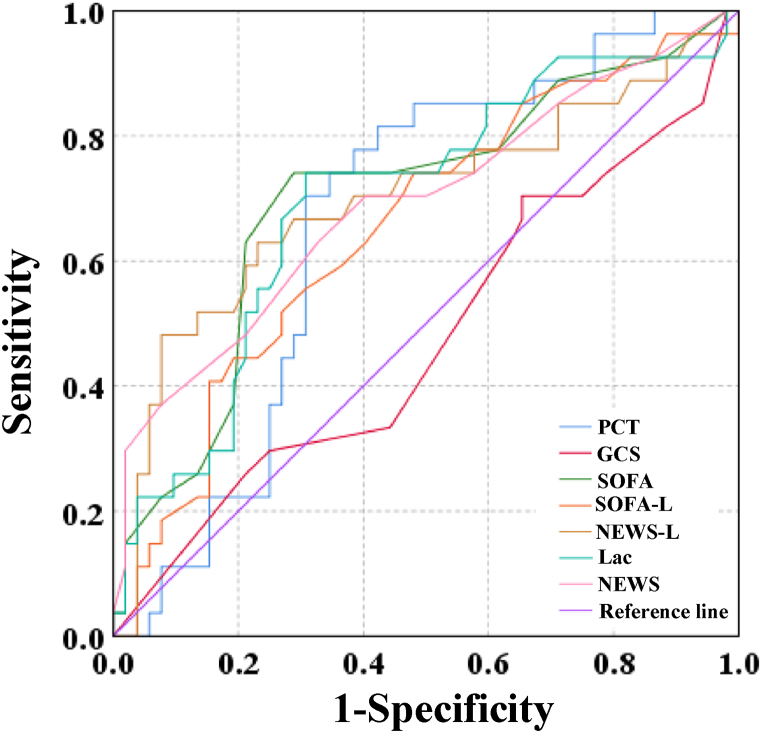
ROC curve of 28-day prognosis evaluated by various indexes alone.

The ROC curves for BLA, PCT, NEWS score, NEWS-L score, GCS score, SOFA score, and SOFA-L score yielded AUC of 0.695, 0.665, 0.692, 0.698, 0.477, 0.700, and 0.653, respectively (Fig. 2). Among these indicators, the SOFA score had the largest AUC, followed by the NEWS-L score and BLA level, while the GCS score had the smallest AUC. The AUC for the GCS score was drastically inferior to that of the other indicators (*P* < 0.05). The AUCs differed slightly among BLA, PCT, NEWS score, NEWS-L score, SOFA score, and SOFA-L score when compared pairwise (*P* > 0.05).

## 4 Discussion

Sepsis is a severe bacterial infection-related disease characterized by systemic inflammatory response syndrome (fever, tachycardia, tachypnea, altered white blood cell count, etc.), circulatory system dysfunction (hypotension, reduced cardiac output, inadequate perfusion, etc.), and organ dysfunction (caused by tissue hypoxia). Affected organs may include the lungs, kidneys, heart, liver, central nervous system, among others [13,14]. Pathogenic bacteria causing sepsis can originate from various sites, such as the lungs, urinary tract, and gastrointestinal tract. Some studies have indicated that rapid identification of the infection site plays a crucial role in selecting appropriate antibiotics, actively controlling infection spread, and reducing mortality rates [15,16]. The etiology and clinical manifestations of sepsis differ depending on the infected site in patients. Pulmonary infections are often accompanied by respiratory distress and cough, while urinary tract infections may cause symptoms such as dysuria and urinary retention [17]. The research findings show that abdominal infections have a significant impact on patient prognosis and can lead to organ dysfunction and even death. In contrast, skin and soft tissue infections are generally more manageable and have a lesser impact on prognosis [18]. The results indicate that respiratory system infections are the most common sites of infection in sepsis, followed by abdominal digestive tract infections and urinary system infections. Among 130 fatal cases, the death rate was as high as 24.21 % (130/537), respiratory system infections and abdominal digestive tract infections were the main causes of death, accounting for 31.97 % and 26.39 %, respectively. There were no deaths reported among patients with infections in other sites, while skin and soft tissue infections and central nervous system infections had few fatalities. When analyzing cases without infection, a mortality rate of 12.50 % was observed. As indicated by a study [19], the most common site of infection in septic patients is the lungs (69.5 %), with a mortality rate as high as 40.3 %, suggesting that respiratory tract infections are one of the primary causes of mortality in sepsis. Both studies underscore the significance of respiratory system infections in sepsis. However, in this study, the sepsis mortality rate of 24.21 % is notably lower than the 40.3 % reported in the referenced study. Analysis suggests that this difference may be attributed to factors such as patient ethnicity and a shorter observation period. Caraballo et al. (2019) [20] found that respiratory system infections were also the most common sites of infection in patients with sepsis, followed by abdominal digestive tract and urinary system infections. The study also revealed a higher correlation between sepsis and cardiovascular diseases, acute kidney injury, and cancer.

The results indicate that the main factors influencing mortality in sepsis include gender, age, mode of admission, treatment upon admission, use of emergency mechanical ventilation, septic shock, administration of vasoactive drugs, heart rate, respiratory rate, SpO_2_, oxygen supplementation, PaO_2_/PAO_2_ ratio, A-aDO_2_, OI, PLT, SCr, BUN, D2 dimer, CRP, PCT, IL-6, ICU hospitalization, total length of hospital stay, ICU/total hospital stay, NEWS score, GCS score, SOFA score, SOFA-L score, NEWS-L score, and lactate level. Current research findings demonstrate that older age, frailty, and immunocompromised individuals have a higher mortality rate after developing sepsis [21]. Some studies have found that male patients are more prone to sepsis and have higher mortality rates compared to female patients [22,23]. This may be related to weaker immune responses to infections in males compared to females [24]. Gender differences exist in hormone levels, chromosomal composition, and immune cell activity, among other factors. These differences may result in a weaker immune response to infections in males compared to females, thereby impacting the intensity and efficacy of immune responses [25]. Additionally, research has shown that female patients tend to have faster recovery after receiving the same treatment [26]. The correlation between mode of admission (self-arrival or ambulance) and mortality in sepsis is a common research topic. Numerous studies have shown that using an ambulance to reach the hospital (i.e., emergency medical services) significantly reduces the risk of sepsis-related mortality upon admission compared to self-arrival [27,28]. Research indicates that invasive mechanical ventilation may increase the risk of death in sepsis patients, while non-invasive ventilation may reduce mortality rates [29]. Furthermore, some studies suggest that early initiation of mechanical ventilation can improve the prognosis of sepsis patients [30,31]. Sepsis patients have a higher probability of developing shock, and the mortality rate is also higher among those with shock [32]. When blood pressure and organ perfusion can't be restored on the basis of fluid resuscitation, vasoactive drugs can be considered to improve blood pressure and tissue perfusion [33]. Common vasoactive drugs are dopamine, dobutamine, and norepinephrine. Nevertheless, other studies have also indicated that the use of vasoactive drugs, under controlled blood pressure, may be beneficial in improving organ function and prognosis [34]. They can assist in the treatment of sepsis by promoting fluid and electrolyte balance, among other aspects. In sepsis patients, a high heart rate may increase the risk of death [35]. Respiratory rate is an important vital sign in sepsis patients, and some research has suggested a potential association between high respiratory rate and increased risk of sepsis-related death [36]. Nevertheless, other researchers have pointed out that there is no notable correlation between high respiratory rate and sepsis mortality [37]. Binary regression analysis results showed neglectable correlation between respiratory rate and time of sepsis-related death. Sepsis is an inflammatory response syndrome. Low blood oxygen saturation has been associated with increased sepsis mortality [38]. Studies have indicated that low SpO_2_ levels are a reliable indicator for predicting mortality in severe sepsis patients [39]. Excessive oxygen supplementation may increase the mortality rate in sepsis patients [40]. Recent studies have suggested that appropriate oxygen supplementation may be beneficial in improving tissue oxygenation and prognosis for certain types of sepsis patients [41,42]. The decrease of arterial oxygen partial pressure (PaO_2_) is a sign of pulmonary dysfunction in septic patients, which is clinically manifested as hyperventilation (increased respiratory frequency), which may lead to low arterial carbon dioxide partial pressure (PaCO_2_) [43]. If metabolic acidosis (lactic acid and/or renal acidosis) still exists, the respiratory alkalosis caused by it can be amplified. Treatment of hypoxemia requires oxygen inhalation, and in the most serious case, mechanical ventilation is needed. The arterial-alveolar oxygen partial pressure ratio (PaO_2_/PAO_2_) is used to predict the oxygen tension in alveoli. At present, studies have shown that the blood lactic acid level of children with sepsis in ICU is positively correlated with PaO_2_. It is suggested that the more severe the degree of hypoxia, the higher the rate of blood lactic acid and lactic acid poisoning, and the worse the prognosis of children [44]. A-aDO_2_ refers to the ratio of arterial oxygen tension (PaO_2_) to the fraction of inspired oxygen (FiO_2_) in arterial blood gas analysis. A retrospective study found that in sepsis patients, a high A-aDO_2_ level was associated with higher mortality rates [45]. Changes in A-aDO_2_ can serve as a prognostic indicator when using mechanical ventilation to treat sepsis patients. The OI is a parameter utilized to evaluate respiratory function and is widely applied in intensive care and mechanical ventilation. Some research results have shown that a low OI (<200) is greatly associated with increased mortality in sepsis patients [46,47], but other studies have found this correlation to be insignificant. In sepsis patients, low PLT levels are associated with poor prognosis. Additionally, studies have shown that excessive platelet transfusion, even within the normal range of platelet count, may increase the mortality rate in patients with sepsis [48]. SCr is a marker utilized to assess renal function, and elevated levels of SCr are associated with an increased risk of sepsis-related mortality. Early renal impairment (at the onset of sepsis) indicated a higher mortality rate. Nevertheless, some studies have found that elevated SCr levels do not necessarily predict higher mortality rates, as other factors may influence this relationship, such as patient age and baseline health status [49]. A retrospective cohort study found that elevated BUN levels were associated with increased mortality in septic patients receiving mechanical ventilation in the intensive care unit [50]. Nevertheless, this correlation is not definitive, and there are other factors that may influence the results, such as age, gender, body mass index, and baseline health status. Elevated levels of D2 dimer can lead to severe inflammatory responses and are associated with sepsis-related mortality. A systematic review and meta-analysis revealed that elevated levels of D2 dimer are significantly associated with an increased risk of sepsis-related mortality, suggesting that D2 dimer can serve as a robust biomarker for sepsis prognosis [51]. Studies have demonstrated that inflammatory markers such as CRP, PCT, and IL-6 are commonly utilized indicators to reflect the degree of inflammatory response in sepsis and predict disease severity. A retrospective study found that when patients had high levels of CRP, PCT, and IL-6, their sepsis mortality rate was relatively higher. Additionally, research has shown that longer hospital stays and extended CCU/total hospital stay durations may increase the risk of mortality in septic patients.

The results suggested that PCT, NEWS score, GCS score, SOFA score, SOFA-L score, NEWS-L score, and BLA were notably correlated with sepsis-related mortality (*P* < 0.001). This indicates that PCT, NEWS score, GCS score, SOFA score, SOFA-L score, NEWS-L score, and BLA can serve as effective reference standards for diagnosing and predicting sepsis-related mortality. These indicators can be utilized to identify high-risk patients and promptly initiate necessary treatment measures to improve their prognosis. BLA is one of the commonly utilized biomarkers in sepsis assessment, and its elevated levels are associated with tissue hypoxia, cellular metabolic dysregulation, and circulatory failure. In clinical practice, BLA can be utilized as a prognostic assessment marker and a monitoring indicator for treatment response. Nevertheless, BLA has limitations such as nonSpe, poor Sen, and susceptibility to various factors. Hence, when using BLA for sepsis assessment, a comprehensive evaluation of the patient's clinical presentation, other biochemical markers, and imaging findings is necessary [52]. The results revealed that the AUC for BLA, PCT, NEWS score, NEWS-L score, GCS score, SOFA score, and SOFA-L score were 0.695, 0.665, 0.692, 0.698, 0.477, 0.700, and 0.653, respectively. The AUC of SOFA score was the largest, followed by NEWS-L score and blood lactic acid level, and the AUC of GCS score was the smallest. The larger the AUC, the higher the accuracy of distinguishing positive and negative results. Among them, the AUC of SOFA score and NEWS-L score was very close, both of which were about 0.699, which shows that both methods can be used to evaluate sepsis and have certain predictive ability. By setting the Cutoff Value, we can choose between different sensitivity and specificity, but generally speaking, the optimal cutoff value may need to be considered more comprehensively when considering the overall disease risk.

## 5 Conclusions

This work aimed to investigate the adoption value of BLA combined with the NEWS in the early screening of sepsis patients and assessing their severity. The results revealed that the use of ventilators, septic shock, PCT level, NEWS score, GCS score, SOFA score, SOFA-L score, NEWS-L score, and BLA level were identified as independent risk factors for predicting the prognosis of sepsis patients. The combination of BLA with NEWS (NEWS-L) score and SOFA score demonstrated certain advantages in assessing the prognosis of sepsis. Nevertheless, this work had geographical limitations in terms of sample inclusion and relied on retrospective analysis of relevant information from electronic medical records, which resulted in missing indicators such as nutritional status. Future work should focus on expanding the sample size to validate the results of this work. In conclusion, this work provides scientific evidence for the early identification and intervention of sepsis, supports the revision of clinical diagnostic guidelines, and offers reference for precise treatment of sepsis.

## Ethics statement

Research experiments conducted in this article with humans were approved by the Ethical Committee and responsible authorities of our research organization(s) following all guidelines, regulations, legal, and ethical standards as required for humans or animals.

## Data availability statement

The data and materials used in this study are available upon request.

## CRediT authorship contribution statement

**Qiyun Gan:** Writing – review & editing, Writing – original draft, Validation, Supervision, Funding acquisition, Formal analysis, Data curation, Conceptualization. **Zhengning Li:** Writing – review & editing, Writing – original draft, Validation, Methodology, Formal analysis, Data curation, Conceptualization. **Xin Li:** Writing – review & editing, Supervision, Resources, Formal analysis, Data curation. **Yinghua Huang:** Writing – review & editing, Writing – original draft, Project administration, Methodology, Investigation, Conceptualization. **Haojian Deng:** Writing – review & editing, Writing – original draft, Supervision, Software, Formal analysis, Data curation, Conceptualization.

## Declaration of competing interest

The authors declare that they have no known competing financial interests or personal relationships that could have appeared to influence the work reported in this paper.
